# The longitudinal association between internet addiction and depressive and anxiety symptoms among Chinese adolescents before and during the COVID-19 pandemic

**DOI:** 10.3389/fpubh.2022.1096660

**Published:** 2023-01-18

**Authors:** Li Zhao, Xiang Li, Qin Yang, Yinhui Peng, Lihua Jiang, Peng Jia, Wei Shi

**Affiliations:** ^1^Department of Health Policy and Management, West China School of Public Health and West China Fourth Hospital, Sichuan University, Chengdu, China; ^2^School of Resource and Environmental Sciences, Wuhan University, Wuhan, China; ^3^International Institute of Spatial Lifecourse Health (ISLE), Wuhan University, Wuhan, China; ^4^Institute for Disaster Management and Reconstruction (IDMR), Sichuan University, Chengdu, China

**Keywords:** internet addiction, depressive, anxiety, adolescents, COVID-19

## Abstract

**Background:**

The COVID-19 pandemic and related prevention policies, such as home quarantine or online courses, could increase the risks of experiencing internet addiction and mental health problems among Chinese adolescents. There is a lack of longitudinal evidence to show the association between internet addiction symptoms and psychological consequences (e.g., depressive and anxiety symptoms).

**Objective:**

This study aimed to explore the association between internet addiction and depressive and anxiety symptoms before and during the coronavirus disease 2019 (COVID-19) pandemic.

**Methods:**

An effective sample of 7,958 Chinese adolescents was recruited for this two-wave longitudinal survey conducted over a six-month interval. All participants completed two-wave surveys before and during the COVID-19 pandemic. A longitudinal cross-lagged path model was used to analyze the associations between internet addiction and depressive and anxiety symptoms after controlling for four covariates (i.e., age, sex, minority, and COVID-19 influence).

**Results:**

Higher depressive and anxiety symptoms before COVID-19 significantly predicted severe internet addiction during COVID-19. Results showed a significant bidirectional relationship between internet addiction and depressive symptoms. Furthermore, the prevalence of internet addiction displayed an increasing trend over the two waves. Conversely, a reduced prevalence of anxiety and depressive symptoms was observed over the two waves.

**Conclusion:**

This current study provided valuable evidence that psychological problems and internet addiction significantly influenced each other before and during the COVID-19 outbreak. Consequently, the presence of psychological problems before and during the COVID-19 outbreak could indicate internet addiction. Thus, depression- and anxiety-related psychotherapies should be developed to prevent internet addiction among Chinese adolescents.

## Introduction

Several coronavirus disease 2019 (COVID-19) prevention and control measures could lead to prolonged internet exposure and exacerbate internet-related addictive behaviors and symptoms among Chinese adolescents (1). Globally, the COVID-19 outbreak resulted in multiple pressure events, including interpersonal relationships, financial difficulty, unemployment, and quarantine policy. Consequently, these events can widely impact the psychological, social, and physical wellbeing of individuals (2). To stem further transmission of COVID-19, a comprehensive lockdown policy was implemented early in 2020 across severely affected cities in mainland China, such as Wuhan, Chengdu, and Shanghai. Furthermore, the Ministry of Education imposed school closures to reduce social interaction and large-scale congregation. All students across the lockdown-implemented cities were required to stay home, maintain social distancing, undergo nucleic acid daily testing, and learn online (3). Therefore, students at home experienced increased exposure to the internet and spent more time engaging daily in online activities (i.e., socializing, chatting, and leisure activities) during the COVID-19 pandemic than before. Consequently, this could increase their risk of suffering from internet addiction symptoms (4).

Internet addiction refers to the excessive, compulsive, or poorly controlled behaviors related to spending plenty of time on internet use that results in psychiatric impairment, dysfunction, and distress (5). Recent research indicated an upsurge in the prevalence of problematic internet use from 14.4% before COVID-19 to 52% during the pandemic (6). Moreover, the latest systematic review analyzed 11 related studies and revealed that during COVID-19 there was an increased dependence on internet use (46.8%) and a low to mild degree of internet addiction was very common (62%) (5). Furthermore, several prior studies with small samplings reported that adolescents were vulnerable to suffering from internet addiction symptoms, and the prevalence of addictive internet use ranged from 24.4 to 55% among Chinese adolescents during COVID-19 (1, 3, 7). Additionally, previous researchers have reported on the exaggerated prevalence of depression (8, 9) and anxiety (10, 11) among the Chinese population during the COVID-19 pandemic. The multiple pathways model effectively supported the close and frequent relationship between depressive and anxiety symptoms in youth (12). The previous review evidenced the interplay between depressive and anxiety symptoms among adolescents (12). However, large-sampling evidence investigating the developing trend in internet addiction, depressive, and anxiety symptoms among Chinese adolescents across the COVID-19 stages (e.g., before and during the COVID-19 outbreak) remains limited. Thus, the following hypotheses were formulated based on prior literature:

H1: Internet addiction among adolescents increased from before COVID-19 to during COVID-19.
H2: Depressive symptoms among adolescents worsened from before COVID-19 to during COVID-19.
H3: Deteriorated anxiety symptoms among adolescents is present from before COVID-19 to during COVID-19.

Many studies have found a significant association between internet addiction and psychological problems among adolescents during COVID-19 (1, 13, 14). Furthermore, a previous study with a sample of 561 adults in Mexico found that internet addiction was significantly associated with depression and anxiety (15). Similarly, Servidio et al. (16) confirmed the significant and positive association between anxiety and internet addiction during the first national COVID-19 lockdown based on a sample of 454 Italian students. A large-scale survey also proved that internet addiction positively correlated with depression among 4,734 Indonesian adults (6). Moreover, previous studies indicated a high rate of co-occurrence between internet addiction and mental health disorders (9, 17). In addition, the previous study emphasized the hazardous consequences underlying the comorbidity of internet addiction and psychological problems, namely, worse prognosis, severe damage to social function, and higher interference with everyday life (17). Furthermore, a few studies indicated that individuals who were younger (15), female (18), and minorities (19), were more impacted by COVID-19 (16) and associated with internet addiction, anxiety, and depressive symptoms. However, most studies exploring this issue only focused on the adult population using a cross-sectional survey design (6, 15, 16). To date, large-sample longitudinal evidence exhibiting the association between internet addiction and psychological problems among large-sample Chinese adolescents remains limited.

Some theories provide conceptual elucidations underscoring the links between internet addiction and mental health problems. For example, the Problem-Behavior Theory proposed by De Leo and Wulfert (20) showed a synthetic theoretic framework illustrating individuals who lack a social network and exhibit worse psychologically internalizing problems (e.g., issues with social anxiety and depression) were more susceptible to problematic Internet use (21). Furthermore, the Interaction of the Person-Affect-Cognition-Execution (I-PACE) model presented the interpretation mechanisms for internet addiction development (22, 23). This model proposed four key variables, namely, personal characteristics, emotional replies for internal or external incentives, executive and repressive control, and resolution-making behavior. Consequently, these variables led to internet-related problematic usage (23). Based on the I-PACE, when people with psychological problems experienced internet usage-related signals, they could be affected by deteriorating negative emotions, possibly resulting in reduced executive and repressive control (22). Therefore, individuals could be seeking mood-dismissing approaches to evade real life and tend to engage in internet-related activities. Conversely, individuals gain happiness from virtual satisfaction and recompense real life experiences through internet usage (24). These theoretical frameworks have been empirically supported for investigating internet addiction and psychological problems (24, 25). Based on previous literature, the following hypotheses were proposed:

H4: Internet addiction before COVID-19 significantly and positively predicts depressive symptoms during COVID-19.
H5: Internet addiction before COVID-19 significantly and positively predicts anxiety symptoms during COVID-19.
H6: Depressive symptoms before COVID-19 significantly and positively predict internet addiction during COVID-19.
H7: Anxiety symptoms before COVID-19 significantly and positively predict internet addiction during COVID-19.

This current study comprised two-wave longitudinal data and utilized the cross-lagged modeling analysis to explore the associations between internet addiction and mental health problems (e.g., depressive and anxiety symptoms) among Chinese adolescents before and during the COVID-19 pandemic. The controlled covariates in this study included age, sex, minority, and COVID-19 influence in the modeling analysis. Furthermore, the prevalence changes in internet addiction, depression, and anxiety symptoms before and during the stages of COVID-19 were examined in this study.

## Methods

### Data collection

The dataset of the current study is derived from the Chengdu Positive Child Development (CPCD) research project (26). The participants were recruited from five primary or middle schools in Chengdu using the simple random sampling approach. The self-report questionnaire was used to collect data before and during COVID-19 in China. Two-wave studies have been completed with six-month time intervals, namely Wave 1 (W1, December 23, 2019, to January 13, 2020, before COVID-19) and Wave 2 (W2, June 16 to July 8, 2020, during COVID-19). Questionnaires were distributed to 10,370 potential participants, and 8,749 valid responses were returned in the W1 study (valid reply rate = 84.37%, 48.38% women). A total of 7,958 participants were followed in the W2 study (valid reply rate = 76.74%). However, there were 791 participants who were lost to be followed in the W2 study. The study was approved by the Research Ethics Committee of the research university and the schools' administrations where participants were recruited (approval number: K2020025). All participants and their guardians were informed of the research objective, procedure, privacy, risk, and data retention before providing their signed informed consent.

## Measures

### Internet addiction

The current study used the Chinese version of the Young Internet Addiction Test (YIAT), which assesses the severity of internet addiction through the 20-item self-report measure (27). Participants were guided to rate each item on a five-point Likert scale ranging from 1 to 5 (1 = rarely and 5 = always). For example, a sample item assessed, “How often do you find that you stay online longer than you intended?” Thereafter, the scores of all items were calculated which displayed the degree of internet addiction symptoms. A higher score denoted severe internet addiction. Based on the recommended diagnostic standards (27), total scores exceeding 31 indicated an abnormal level of Internet use. Furthermore, YIAT showed significant reliability and validity in previous research (27). Similarly, the reliabilities of this scale were excellent in both W1 (Cronbach's alpha = 0.93) and W2 (Cronbach's alpha = 0.94) surveys. The construct validities of this scale were good in both W1 (Kaiser-Meyer-Olkin [KMO] measure of sampling adequacy = 0.87) and W2 (KMO measure of sampling adequacy = 0.87) surveys.

### Anxiety symptom

The Chinese version of the Screen for Child Anxiety Related Emotional Disorders (SCARED) subscale assessed the anxiety symptom for the last 3 months (28). A three-point Likert scale was used in the nine-item subscale, rating from 0 = never to 2 = often. One sample item is “people tell me that I worry too much.” Adding the scores of all items denoted the severity of anxiety symptoms. A higher score represented a more serious anxiety symptom. The cut-off score was recommended as 9 for diagnosing anxiety disorders among adolescents (28). Previous studies evidenced the great validity and reliability of SCARED that applied in this current study (28). The reliabilities of this scale were good in the W1 (Cronbach's alpha = 0.86) and W2 (Cronbach's alpha = 0.88) surveys. This scale showed the excellent construct validities in both W1 (KMO measure of sampling adequacy = 0.92) and W2 (KMO measure of sampling adequacy = 0.93) surveys.

### Depressive symptom

The Chinese Version of the Center for Epidemiologic Studies Depression Scale (CES-D) consists of 20 items that evaluated the degree of depressive symptoms during the past week (29). A four-point Likert scale was used to rate each item (0 = not at all, 3 = often). Furthermore, there were four reversed score items, namely, the fourth, eighth, twelfth, and sixth items. A sample item states, “I experienced difficulty with focusing my mind on what I was doing.” Similarly, this scoring method followed the previously suggested rules that summed the scores of all items. Consequently, higher scores represented worse depressive symptoms (29). Moreover, total scores that were higher than 15 represented a depression diagnosis. Previous research found that the CES-D exhibited great reliability and validity (29). In the current study, the CES-D showed decent reliabilities in both W1 (Cronbach's alpha = 0.87) and W2 (Cronbach's alpha = 0.89) surveys. The construct validities of this scale were excellent in both W1 (KMO measure of sampling adequacy = 0.94) and W2 (KMO measure of sampling adequacy = 0.95) surveys.

### Covariates

The covariates were informed by the demographics (i.e., age, sex, and minority) and COVID-19 influence. The current study defined COVID-19 influence as the perceived degree of personal influence resulting from COVID-19 in terms of daily life and social activities. Based on the COVID-19 pandemic context, nine items were designed to assess the COVID-19 influence on sampling participants (30). COVID-19 influence was evaluated in three ways. First, four items assessed perceived seriousness, threat, contagion danger, and precaution against COVID-19 using a four-point Likert scale (1 = not at all, 4 = extremely severe). Second, one dichotomous question asked whether the COVID-19 case was confirmed in participants or their families (1 = no, 2 = yes). Finally, four questions investigated the perceived effect of COVID-19 on diet, daily study, interpersonal relationships, and leisure activities ranging from 1 to 4 (1 = none to 4 = extremely influenced). The level of COVID-19 influencing participants was determined by summing the scores of all items. Consequently, a higher score represented a more severe perceived influence of COVID-19.

### Statistical analysis

The data analysis contained fivefold processes and was applied using computer software, AMOS Version 23 and SPSS version 24 (31). First, descriptive analysis was used to explore the participants' features based on the study variables (i.e., age, sex, minority, COVID-19 influence, internet addiction, depressive, and anxiety symptoms). Second, the Independent-Samples t-tests and Pearson's chi-squared tests were applied to analyze the significant differences between study variables across study waves. Third, a correlation matrix was used to test the significant links between study variables in the condition of controlling and without controlling covariates. Fourth, an invariance measurement test was used to assess the stability of study variables in a longitudinal cross-lagged model. The multi-collinearity test evaluated the risk of collinearity issues among study variables. Finally, a longitudinal cross-lagged model was constructed and examined using structural equation modeling. Consequently, three significant levels were used in this current study, including *p* < 0.05, *p* < 0.01, and *p* < 0.001 (32).

## Results

### Participant characteristics

The final sampling participants consisted of 7,958 Chinese adolescents who completed the two-wave surveys. The study comprised 4,112 men (51.7%) and 3,846 (48.3%) women, with a mean age of 12 years old (standard deviation [SD] = 2.15, age ranging from 7 to 17). Most participants belong to the Han ethnic group (*n* = 7,893, 99.18%). The current study did not find any significant differences between valid samples and missing track samples in all study variables (*p*s > 0.05), apart from internet addiction, anxiety, depressive symptoms, and age. Furthermore, older participants with more severe internet addiction, higher anxiety, and more serious depressive symptoms were more likely to drop out of the follow-up survey (Table 1).

**Table 1:** Participants' characteristics (*n* = 7,958).

**Variables**	**(M, SD, *N*, %)**
Age (7–17)^***^	M = 12, SD = 2.15
**Sex**	
Men	4,112 (51.7%)
Women	3,846 (48.3%)
Minority	
Hans	7,893 (99.2%)
Non-Hans	65 (0.8%)
COVID-19 influence	M = 21, SD = 4.05
**Internet addiction** ^***^	
Wave 1	M = 34.66, SD = 14.76
	N_Yes_ = 3,931 (49.40%)
	N_no_ = 4,027 (50.60%)
Wave 2	M = 35.06, SD = 15.13
	N_yes_ = 4,016 (50.46%)
	N_no_ = 3,942 (49.54%)
**Depressive symptom**	
Wave 1	M = 14.40, SD = 10.16
	N_Yes_ = 3,078 (38.68%)
	N_no_ = 4,880 (61.32%)
Wave 2	M = 14.36, SD = 10.62
	N_Yes_=2,924 (36.74%)
	N_no_=5,034 (63.26%)
**Anxiety symptom** ^***^	
Wave 1	M = 3.71, SD = 3.96
	N_Yes_ = 1,036 (13.02%)
	N_no_ = 6,922 (86.98%)
Wave 2	M = 3.33, SD = 4.07
	N_Yes_ = 1,016 (12.77%)
	N_no_ = 6,942(87.23%)

(1) SD, Standard Deviation; M, Mean; N_yes_, number of participants with Internet addiction, anxiety or depressive symptoms; N_no_, number of participants without Internet addiction, anxiety or depressive symptoms; (2) Diagnostic cut-off scores: Internet addiction≥31, depressive symptom≥15, anxiety symptom≥9; (3)

*** p < 0.001 (chi-square tests or t-tests across wave data).

### Inter-correlations analysis

Table 2 depicts the means, inter-correlation, and the SD results among the study variables. According to recommended criteria for the correlation degree (33), the absolute coefficients value between 0.50 and 1 represent a high level of correlation, while the values 0.30 and 0.49 represent a moderate level of correlation, and the values below 0.29 indicate a low level of correlation. When controlling for the four covariates of age, sex, minority, and COVID-19 influence, most variables presented high or moderate correlated links with each other, except W1 internet addiction with W2 depressive (r = 0.27, *p* < 0.001) and W2 anxiety symptoms (r = 0.24, *p* < 0.001). Relatively low degrees of correlation between the remaining study variables were shown. Likewise, upon removing the controlled four covariates, the correlations of W1 internet addiction with W2 depressive (r = 0.29, *p* < 0.001) and W2 anxiety symptoms (r = 0.27, *p* < 0.001) remained at a significantly and relatively low level. Moreover, internet addiction displayed significant and positively longitudinal correlations with depressive and anxiety symptoms across the study waves.

**Table 2:** Inter-correlations between Internet addiction, depressive and anxiety symptoms (*n* = 7,958).

**#**	**Variables**	**1**	**2**	**3**	**4**	**5**	**6**
1	W1 anxiety symptom	/	0.49^***^	0.61^***^	0.41^***^	0.38^***^	0.31^***^
2	W2 anxiety symptom	0.52^***^	/	0.40^***^	0.62^***^	0.24^***^	0.38^***^
3	W1 depressive symptom	0.61^***^	0.41^***^	/	0.52^***^	0.40^***^	0.33^***^
4	W2 depressive symptom	0.43^***^	0.64^***^	0.53^***^	/	0.27^***^	0.41^***^
5	W1 internet addiction	0.39^***^	0.27^***^	0.38^***^	0.29^***^	/	0.50^***^
6	W2 internet addiction	0.33^***^	0.41^***^	0.32^***^	0.43^***^	0.57^***^	/

(1) The number in lower left is the correlation coefficient without controlling for the four covariates (i.e., age, sex, minority, and COVID-19 influence). The number in top right is the correlation coefficient after controlling four covariates. (2)

*** p < 0.001. (3) Correlation coefficients interval: High level: between ± 0.50 and ± 1; Moderate level: between ± 0.30 and ± 0.49; Low level: below + 0.29.

### Invariance measurement

Based on the previous recommendations, stability coefficients were selected to evaluate the invariance measurement of study variables across waves (34). When the stability coefficients were higher than 0.4, the invariance measurement was statistically supported in the longitudinal cross-lagged model. Consequently, this study found that stability coefficients of internet addiction as well as anxiety and depressive symptoms at both waves were 0.62, 0.59, and 0.50, respectively. Thus, this result supported the invariance measurement in the longitudinal cross-lagged path model.

### Multi-collinearity test

As suggested by previous research, a multi-collinearity issue could occur when the values of variance inflation factor (VIF) and Tolerance were higher than 4.0 and lower than 0.25, respectively. Furthermore, the results of multi-collinearity test displayed that internet addiction (VIF = 1.23, Tolerance = 0.82), depressive (VIF = 1.65, Tolerance = 0.61), and anxiety (VIF = 1.66, Tolerance = 0.60) symptoms did not lie in the boundaries. Consequently, a very low risk of multi-collinearity was found in the current study.

### Prevalence of internet addiction, depressive and anxiety symptoms

Table 1 displays the prevalence of internet addiction, depressive, and anxiety symptoms in both study waves. First, the prevalence of internet addiction increased from 49.40% in W1 to 50.60% in W2. Conversely, the prevalence of anxiety symptoms decreased from 13.02% at W1 to 12.77% at W2. Similarly, the prevalence of depressive symptoms declined from 38.68% at W1 to 36.74% at W2. Furthermore, the results of the paired-sample *t*-test yielded significant differences in the mean level of internet addiction and anxiety symptoms across study waves (*ps* < 0.001). These results supported H1 but rejected H2 and H3.

### Cross-lagged path model

Based on previous research (35), a variety of goodness-of-fit indices were suggested for use to appraise the overall model quality and paths significance, including χ^2^/df (degree of freedom), Standardized Root Mean Square Residual (SRMR), Root Mean Square Error of Approximation (RMSEA), Comparative-Fit Index (CFI), Normed Fit Index (NFI), and Tucker and Lewis Index (TLI). Consequently, when the χ^2^/df was lower than 5.0, CFI, TLI, and NFI were higher than 0.95. Furthermore, RMSEA and SRMR were smaller than 0.05. Thus, the longitudinal cross-lagged path model was good. Moreover, the model indicated the wellness of the proposed model reproducing the observed longitudinal data.

**Figure 1:**
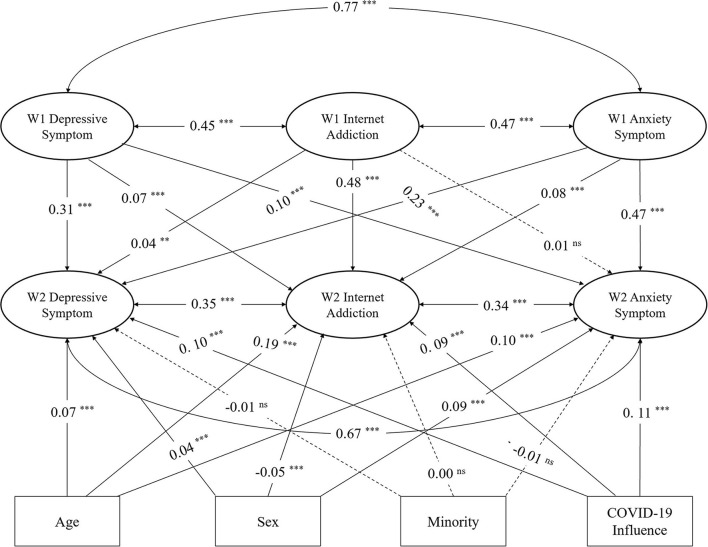
The longitudinal cross-lagged model with bidirectional effects between internet addiction, depressive, and anxiety symptoms (*n* = 7,958). (1) Dotted lines represent insignificant paths, ns = not significant. (2) **p < 0.01, ***p < 0.001.

The longitudinal cross-lagged model exhibited the great goodness-fit indexes without controlling for the four covariates, χ^2^ (2075) = 9695.001, *p* < 0.001, χ^2^ /df = 4.672, CFI = 0.973, NFI = 0.966, TLI = 0.970, RMSEA = 0.021, and SRMR = 0.031. Furthermore, the model fit was good after controlling for these four covariates, χ^2^ (2323) = 11221.203, p < 0.001, χ^2^ /df = 4.830, CFI = 0.969, NFI = 0.961, TLI = 0.966, RMSEA = 0.022, SRMR = 0.031. Moreover, Figure 1 illustrates the results of concurrent relations between study variables. Consequently, this study found significant concurrent associations between internet addiction and depressive symptoms in W1 (r = 0.45, SE = 0.022, *p* < 0.001) and W2 (r = 0.35, SE = 0.013, *p* < 0.001). Internet addiction also showed significantly co-instantaneous links with anxiety symptoms in W1 (r = 0.47, SE = 0.016, *p* < 0.001) and W2 (r = 0.34, SE = 0.012, *p* < 0.001). Thus, this study found significant co-current links between depressive and anxiety symptoms in both W1 (r = 0.77, SE = 0.005, *p* < 0.001) and W2 (r = 0.67, SE = 0.003, *p* < 0.001).

Figure 1 represents the cross-lagged model analysis, which suggests that internet addiction in W1 significantly and positively predicts internet addiction (β = 0.48, SE = 0.002, *p* < 0.001) and depressive symptoms (β = 0.04, SE = 0.003, *p* = 0.002) in W2. However, the model was not significant with anxiety symptoms in W2 (β = 0.01, SE = 0.002, *p* = 0.365). Furthermore, depressive symptoms in W1 significantly and positively predicted depressive symptoms (β = 0.31, SE = 0.016, *p* < 0.001), internet addiction (β = 0.07, SE = 0.091, *p* < 0.001), and anxiety symptoms (β = 0.10, SE = 0.015, *p* < 0.001) in W2. Moreover, anxiety symptoms in W1 had significant and positive predictive links with anxiety symptoms (β = 0.47, SE = 0.025, *p* < 0.001), internet addiction (β = 0.08, SE = 0.145, *p* < 0.001), and depressive symptoms (β = 0.23, SE = 0.025, *p* < 0.001) in W2. Consequently, these results supported H4, H6, and H7 but rejected H5.

## Discussion

Based on the cross-lagged path model, this study provided further insight into the concurrent and longitudinal association between internet addiction and symptoms of anxiety and depression. Furthermore, the current study examined the developing trend of several study variables among Chinese adolescents before and during the COVID-19 pandemic. Based on existing knowledge, this study comprises the first large-scale longitudinal study to explore these issues in China. These findings could contribute critical suggestions to elucidate the three inter-related study variables. Moreover, the results can inform program designs underlying internet addiction prevention aimed at Chinese adolescents exposed to COVID-19.

The study findings indicated that the existence of severe depressive symptoms before COVID-19 significantly influenced the severity of internet addiction during the pandemic. This result was consistent with most previous research that found significant and positive relations between depressive disorders and internet addiction (36). For example, a systematic review analyzed 18 related studies to investigate the positive links between internet addiction and depressive disorders (36). Furthermore, some studies supported that depression positively predicted internet addiction in Asian regions (37–39). These findings have been explained in previous studies. For example, adolescents with depressive symptoms usually exhibited a persistent low mood, feeling of sadness, lack of belongingness, and loss of interest in real life (39). Moreover, the Internet provided a virtual world to adolescent users, in which adolescents with depression could access supportive social networks and the pleasure of control while ignoring emotional difficulty and escaping from reality. Furthermore, a study reasonably suggested that adolescents with higher depressive symptoms were more likely to have more severe internet addiction (36). Additionally, the “short” alleles of the serotonin-transporter-linked promoter region (5-HTTLPR) were significantly and simultaneously linked with depressive disorders (40) and internet addiction (41). Moreover, the 5HTTLPR gene was closely related to serotonin function suggesting that the biological weakness in both depressive symptoms and internet addiction could be associated with serotonin dysfunction (41). In turn, this could explain the results discovered in the current study. Therefore, future studies should focus on reducing depressive symptoms that could serve as a precautionary approach to internet addiction and evaluating the depressive degree among adolescents with internet addiction.

Consistent with previous studies, the current study found that severe pre-existing anxiety symptoms before the COVID-19 pandemic significantly predicted a higher level of internet addiction during COVID-19 among adolescents (39, 42, 43). For example, a longitudinal study indicated that anxiety symptoms positively predicted the emergence of internet addiction in the two-year prospective follow-up survey (44). Furthermore, Yayan et al. (45) found that adolescents with problematic symptoms of internet usage had inferior interpersonal relationships or anxious social phobias in the real world. Thus, adolescents with symptoms of social phobia tried to evade the anxiety emerging from face-to-face communication with people through the internet which could provide virtual social support (36). Other research reported that adolescents experienced mentally internalizing issues (e.g., anxious symptoms) when they suffered from the social interaction dilemma in real life (46). Adolescents tended to use the internet (i.e., entertaining activities, relaxing social networks, and anonymously abreacting vents) to mitigate internal mental problems such as anxiety (47). Consequently, this explains the positive association between anxiety and internet addiction found in the current study. Potentially, these findings suggest that social interaction mediated or moderated the association between anxiety symptoms and internet addiction. Thus, future studies should investigate this mechanism further through a longer-term follow-up. Simultaneously, social anxiety-related intervention should be enhanced further when conducting clinical psychotherapy for internet addiction.

Interestingly, the present study demonstrated that internet addiction before COVID-19 was a positive predictor of depressive symptoms but did not significantly predict anxiety symptoms during COVID-19. Previous studies have proved the significant relationship between depression and Internet addiction (36, 37). People tended to excessively use the internet as a dysfunctional handling approach to alleviate negative emotional symptoms such as depression during COVID-19. Similarly, people exposed to internet overuse could suffer from more intrapersonal conflicts in the real world (37). Consequently, this finding indicated a vicious cycle in which depressive symptoms and internet addiction disorder upsurge each other (16). Thus, future studies should provide further insight into the mechanism underlying internet addiction and comorbidity properties with depressive symptoms as this could benefit the development of internet addiction interventions. In contrast, most previous research found that internet addiction was significantly associated with anxiety (14, 48, 49). Furthermore, previous research has provided possible explanations for this result. For example, internet addiction was probably not the priority factor directly resulting in anxious emotions among adolescents during COVID-19 (16). Particularly, the study found that the fear of COVID-19 significantly mediated the association between anxiety symptoms and internet addiction disorder (16). Moreover, a persistently increasing tendency to other daily life concerns during the COVID-19 pandemic was found, namely, psychical health insecurity, family economic pressure, quarantine fear, and academic future uncertainty. Thus, other factors could be more influential and predictive in the context of anxiety symptoms among adolescents over internet addiction.

Previous literature provided evidence that internet addiction was identified as a significant predictor of subsequent depressive symptoms, but was not significant in the anxiety symptoms. Based on the perspectives of biological genes, neurosciences, and society, many previous psychiatric studies consistently evidenced the predictive association between internet addiction and major depressive symptoms (36, 37, 40, 41). For example, prior research indicated the predictive links between internet addiction and depression disorders when controlling other demographics (e.g., sex, age, and school) among adolescents in Taiwan (50). In addition, whether internet usage is significantly linked with anxiety is still inconclusive among adolescents (10, 11, 51). For example, some research found significant links between internet addiction and concomitant anxiety disorders (48, 49). However, some studies reported an insignificantly predictive association between social anxiety and internet addiction when controlling for depression and attention deficit hyperactivity disorder (44). Another previous research included 59 students with internet addiction symptoms and found depressive symptoms were increasing, but their phobic anxiety did not show any changes in the study period (52). These findings might indicate that other psychiatric factors could be more vital predictors of anxiety symptoms than internet addiction among adolescents during COVID-19. In any case, the predictive mechanisms between internet addiction and anxiety symptoms deserve further attention in different contexts in the future.

This current study demonstrated a decreased trend in depressive and anxiety symptoms but an increased tendency in internet addiction from before COVID-19 to during COVID-19. The rising trend in internet addiction was consistent with previous studies on COVID-19 (4, 6). However, the results demonstrating deteriorated psychological problems were inconsistent with many previous studies (11, 53, 54). Possibly, these results occurred because adolescents could spend more time on internet usage during home quarantine and online studies imposed by the COVID-19 pandemic. Consequently, this could increase the risk of experiencing internet addiction (5). Furthermore, increased time for adolescents to stay with their families could improve family support and thus remit mental problems such as depressive and anxiety symptoms among Chinese adolescents during COVID-19 (5). Additionally, there are two possible reasons why anxiety symptoms were reduced during the COVID-19 compared prior COVID-19 pandemic. First, some positive factors that are usually provided to alleviate anxiety symptoms could be suddenly present or more accessible during the COVID-19 lockdown (e.g., more family time and siblings' support) (55). Second, some negative factors that could arouse anxiety symptoms before this pandemic were not as strong or did not present (e.g., school work, interpersonal relationships, and academic stress) (55, 56). Moreover, several studies reported on the diminished prevalence of mental disorders among adolescents exposed to COVID-19 (55–57). Thus, future studies should conduct a study with longer-term follow-up to track the development of internet addiction as well as depression and anxiety symptoms. Particularly, increased psychological intervention should be provided to adolescents to prevent internet-based addictive behaviors during COVID-19.

Another notable finding was that anxiety levels significantly decreased but depression levels did not significantly decline across the two study waves. One possible explanation for this result was that the lockdown policy could bring different influences on depressive and anxiety symptoms among Chinese adolescent students during COVID-19 (55). The lockdown policy made adolescents study at home, which could effectively decrease the anxious symptoms resulting from offline study and interpersonal relationships on campus (55). However, the discordant family atmosphere could be adverse to the remission of depressive symptoms when adolescents have to stay at home during COVID-19 pandemic (5). A future study was suggested to further track different development trends and key influence factors in depressive and anxiety symptoms among Chinese adolescents exposed to COVID-19. Additionally, consistent with some prior studies (16, 36), the results revealed the bidirectional and positive links between internet addiction, depressive and anxiety symptoms in each wave survey. This indicated the possible coexistence of psychiatric problems (e.g., depression and anxiety) and Internet addiction (36). These possible coexisting psychological problems should be paid more attention when treating adolescents with internet addiction symptoms.

### Limitations

The current study had several limitations. First, a self-report questionnaire was used for data collection. Furthermore, the symptoms of depression, anxiety, and internet addiction depended on the scale screening, rather than a clinical diagnosis. Consequently, the scale could influence the accuracy of the prevalence of mental disorders. Thus, future studies should suggest a mixed method design (i.e., qualitative and quotative studies) and select more clinical diagnosis tools for mental disorder prevalence screening. Second, this study did not include all mental factors influencing internet addiction identified in prior literature because of the limited questionnaire length. Thus, future studies could further explore the association between internet addiction and trauma-related disorders, such as acute stress disorder, post-traumatic stress disorder, and adjustment disorder using a longitudinal survey. Third, the effects of the COVID-19 pandemic were controlled for assessing the links between internet addiction, anxiety, and depressive symptoms. However, it should be noted that all samplings were exposed to the COVID-19 pandemic. Thus, the effects of mental health disorders could not be completely detached from the influences of the comprehensive exposure to this pandemic. Fourth, there was a relatively large correlation between depressive and anxiety symptoms in this current study. When two highly correlated symptoms are included in one cross-lagged model, the results could be influenced. It was suggested to combine both symptoms as psychological distress or separate them as depression and anxiety symptom models to further explore this issue in the future (58). Finally, the current data only included two waves, namely, before and during COVID-19. Thus, future studies should exhibit the different relationships between the study variables using longer-term follow-up surveys to further demonstrate these robust links.

## Conclusion

Despite the study limitations, the current study contributed new knowledge to prior theoretical and empirical exploration of the association between internet addiction, anxiety, and depressive symptoms. These findings emphasized that the psychological status before COVID-19 serves as a potential vital signal for internet addiction among adolescents during this pandemic and extended the explanation power of the Problem-Behavior Theory and the I-PACE model for internet-related addictive behavior. Considering the proposed intervention, future studies should examine the longer-term mental health consequences of COVID-19 to improve the early warning of internet addiction among adolescents in the context of a public health emergency. Moreover, internet-related clinical psychotherapeutic efforts should focus on the remission of depressive symptoms among Chinese adolescents during a pandemic.

## Data availability statement

The data that support the findings of this study are available from the corresponding author upon reasonable request.

## Ethics statement

The studies involving human participants were reviewed and approved by the Research Ethics Committee of the University (Approval No. K2020025). Written informed consent to participate in this study was provided by the participants' legal guardian/next of kin.

## Author contributions

LZ did study design and data collection. WS analyzed data, drafted, and submitted this manuscript together. LZ, XL, YP, and QY revised the manuscript. All authors contributed to manuscript checking and approval the final manuscript.
